# Sleep Hygiene Pattern and Behaviors and Related Factors among General Population in West Of Iran

**DOI:** 10.5539/gjhs.v8n8p114

**Published:** 2015-12-17

**Authors:** Habibolah Khazaie, Azita Chehri, Kheirollah Sadeghi, Fatemeh Heydarpour, Akram Soleimani, Zahra Rezaei

**Affiliations:** 1 Sleep Disorders Research Center, Kermanshah University of Medical Sciences, Kermanshah, Iran; 2 Department of Psychology, Kermanshah Branch, Islamic Azad University, Kermanshah, Iran; 3 Department of Clinical Psychology, Faculty of Medicine, Kermanshah University of Medical Sciences, Kermanshah, Iran; 4 Ph.D. Student of Epidemiology, Emam Ali Hospital, Kermanshah University of Medical Sciences, Kermanshah, Iran; 5 Student’s Research Committee, Kermanshah University of Medical Sciences, Kermanshah, Iran; 6 Department of Educational Sciences, Kermanshah Branch, Islamic Azad University, Kermanshah, Iran

**Keywords:** Iran, Kermanshah, sleep behaviors, sleep hygiene

## Abstract

**Introduction:**

Sleep hygiene was found as an important predictor for sleep quality. People’s sleep hygiene can have a major role in their daily function. The purpose of the study was to determine sleep hygiene patterns and sleep hygiene behaviors and factors affecting them in the general population of Kermanshah, Iran.

**Material and methods:**

In this cross-sectional study, 1829 men and 1262 women were selected randomly from 50 clusters of different parts of the city. The inclusion criteria were age between 12 and 65 years and living in Kermanshah. The exclusion criteria were psychiatric disorder and known general medical conditions that affecting sleep. The data collection instruments were demographic questionnaire and Sleep Hygiene Questionnaire, consisted of 13 items about biological rhythm and bed room environment and behaviors that affecting sleep. Data were analyzed by using SPSS version 16 software.

**Results:**

The highest percentage was obtained for irregular woke and went up from day to day or at weekend and holidays (74.8%). Only 213 (6.9%) participants were classified as having good sleep hygiene (score 12-14). The mean age of very poor, poor, moderate, and good sleepers was 34.8 ± 14.4, 33.7 ± 17.4, 36.5 ± 13.8, and 35 ± 13.7years, respectively. There were significant differences between the age of poor and moderate sleepers and also sleep hygiene patterns with respect to sex, education level and job.

**Conclusion:**

Poor sleep hygiene were more frequent in Iranian peoples and the major problem in sleep hygiene in our study was inappropriate sleep schedule.

## 1. Introduction

Sleep a basic need, has an important effect on health (Baldwin et al., 2001; Gottlieb et al., 2006; Gottlieb et al., 2010; Kapur et al., 2002; Masa, Rubio, & Findley, 2000; Meier-Ewert et al., 2004; Walia et al., 2014). Good sleep quality and restful sleep is needed for a good and healthy life (Ozdemir, Boysan, Selvi, Yildirim, & Yilmaz, 2015). Sleep hygiene refers to the lifestyle behaviors and environmental conditions that facilitate sleep and improve sleep quality (American Academy of Sleep Medicine, 2005). Sleep hygiene was originally developed for the treatment of insomnia. In the recent years, there has been increased attention concerning poor sleep hygiene and habits in the general population as well (Brown, Buboltz, & Soper, 2002).

According to the *International Classification of Sleep Disorders* (ICSD2: American Academy of Sleep Medicine), inadequate sleep hygiene behaviors are divided into five categories: inappropriate sleep schedule (e.g., napping and instability bed and wake times), the use of sleep disrupting products (e.g., caffeine and cigarettes), mentally stimulating activities close to bedtime (e.g., planning and involvement in exciting or emotionally upsetting work), involvement in activities other than sleeping in the bed room (e.g., watching television and reading), and failure to sustain a relaxed sleeping environment (e.g., uncomfortable mattress or temperature) (American Academy of Sleep Medicine, 2005).

Many studies have shown that the sleep hygiene behaviors listed above disturb sleep and may be important causes of insomnia (Stepanski & Wyatt, 2003; Jefferson et al., 2005). According to the ICSD**, inadequate sleep hygiene is defined as a sleep disorder because of daily lifestyle activities that are inconsistent with the maintenance of good sleep quality (American Academy of Sleep Medicine, 2005).

According to the National Sleep Foundation, poor sleep quality and quantity is linked to a wide range of health problems, such as obesity, diabetes, hypertension, and depression (National sleep foundation, 2015). Sleep hygiene was found as an important predictor of sleep quality in Italian and American adolescents (Le Bourgeois, Giannotti, Cortesi, Wolfson, & Harsh, 2005). In addition, sleep hygiene factors have a large effect on the prediction of insomnia severity (Gellis, Park, Stotsky, & Taylor, 2014).

However, no previous studies have examined sleep hygiene practices in a large community based on a sample of the general population in Iran. Because people’s sleep hygiene can have a major role in their daily function, the purpose of the current study was to determine sleep hygiene patterns and sleep hygiene behaviors and factors affecting them in the general population of Kermanshah, Iran.

## 2. Material and Methods

In the current descriptive and analytical design (cross-sectional) study, 3,091 people (1,829 (59.2%) men and 1,262 (40.8%) women) from the general population were included. The mean age of the participants was 34.7 ± 14 years. According to the statistical information from the Kermanshah Health Center, the participants were randomly selected from 50 clusters of different parts of the city; 60-70 people from each cluster were selected randomly. A written consent form was completed by all the participants. The inclusion criteria were as follows: age between 12 and 65 years and living in Kermanshah. The exclusion criteria were history of psychiatric disorders and medical condition that affecting sleep.

The data collection instrument was a two-section questionnaire. The first section consisted of seven questions regarding demographics and the second section consisted of 13 items about biological rhythm and bed room environment and behaviors that affecting sleep. We named this questionnaire as Sleep Hygiene Questionnaire (SHQ). This questionnaire consisted of 13 items about biological rhythm and bed room environment and behaviors that affecting sleep. The items of SHQ were derived from the diagnostic criteria for inadequate sleep hygiene as defined in the *International Classification of Sleep Disorders* and other textbooks about sleep disorders.

To determine the scientific validity of the questionnaire, content validity was assessed. For this purpose, the questionnaire was distributed to 10 faculty members of psychiatry at the Medicine School of Kermanshah and was approved after evaluating the comments.

Approximately 10% of the participants retook the questionnaire after a 4-6 week (median 31 days) interval in order to measure test-retest reliability. Spearman test showed that test-retest scores were considerably correlated (0.8).

Data were collected by the students of psychiatry research at the Medical School of Kermanshah who were familiar with the study protocol. The sampling took 2 months and the data were analyzed by using SPSS version 16 software. The test results of SHQ are presented in a term quartile system, which means that frequency distributions are divided into equal fourths. One-quarter (25%) of the cases fall below Q1 and are considered as subjects with very poor sleep hygiene (their sleep hygiene questionnaire scores were between 0 and 6).

The interquartile range, which is an interval from Q1 to Q3, is bounded by the range of scores that represents the middle 50% of the distribution. We divided them into two quartiles, with an interval from Q1 to Q2 (sleep hygiene questionnaire scores ranged between 7 and 9) representing subjects with poor sleep hygiene and an interval from Q2 to Q3 (sleep hygiene questionnaire scores fell between 10 and 11) representing subjects with moderate sleep hygiene.

Finally, one-quarter (25%) of the cases were above Q3 and are considered as subjects with good sleep hygiene (sleep hygiene questionnaire scores fell between 12 and 14).

## 3. Results

The frequency and percentages of all the research variables are shown in Table 1. Results indicate that the highest and lowest percentages were obtained for irregular woke and went up from day to day or at weekend and holidays (74.8%) and using cigarette/tobacco before sleeping (14.3%), respectively.

**Table 1:** Frequency and percent of all study variables

Variable	N	%	Response rate
1- Take daytime naps lasting two hours			
Yes	1859	71	85
No	760	29
2- woke and went up at approximately the same time from day to day or weakened and holidays			
Yes	772	25.2	99
No	2289	74.8
3- Drank caffeinated beverages			
Yes	668	21.8	99.3
No	2400	78.2
4- Used cigarette/tobacco			
Yes	439	14.3	99.2
No	2627	85.7
5- Exercised within 4 hours of bed time (to the point of sweating)			
Yes	582	19	99.1
No	2481	81
6- Worried, planned, or thought about important matters and daily activities			
Yes	1264	41.5	98.5
No	1780	58.5
7- Read in bed			
Yes	1088	35.6	98.9
No	1968	64.4
8-Watched TV in bed			
Yes	1698	55.3	99.9
No	1371	44.7
9- Talked on the phone			
Yes	1021	33.3	99.2
No	2044	66.7
10- Slept in a room with un comfortable temperature, noisy environment and bright room			
Yes	671	21.8	99.5
No	2403	78.2
11- woke and went up at approximately the same time in weekend and holiday			
Yes	1372	44.8	99.2
No	1693	55.2
12- Having a regular exercise program during the week			
Yes	1199	39.3	98.8
No	1855	60.7
13- Watched clock and worried about time to sleep in bed			
Yes	1230	40	99.4
No	1842	60
14- Ate heavy dinner (within 2 hours of going to bed)			
Yes	735	23.9	99.5
No	2339	76.1	

1984(64.2%) participants were classified as having very poor sleep hygiene (score 0-6) or poor sleep hygiene (score 7-9), and 213(6.9%) were classified as having good sleep hygiene (score 12-14) (Table 2). The mean age of very poor, poor, moderate, and good sleepers was 34.8 ± 14.4, 33.7 ± 17.4, 36.5 ± 13.8, and 35 ± 13.7 years, respectively. There was a significant difference between the age of poor and moderate sleepers. There was also a significant difference in sleep hygiene patterns with respect to sex, education level, and job (Table 3).

**Table 2:** Frequency and percent of sleep hygiene according to 4 categories of the study

	N	%
Very poor	529	17.1
Poor	1455	47.1
Moderate	894	28.9
Good	213	6.9
Tot	3091	100

**Table 3:** Uni - variant analysis of effective factor on sleep hygiene

Sleep hygiene												
factors	Very poor		poor		moderate		good						P value
	N	%	N	%	N	%	N	%	Tot	%			
Gender											
Male	326	17.8	826	45.2	562	30.7	115	6.3	1829	100			0.007
Female	199	15.9	627	50	332	26.5	96	7.7	1254	100			
Total	527	17	1453	47.1	894	29	211	6.8	3083	100			
Education												
Under diploma	163	20.1	381	47.1	218	26.9	47	5.8	809	100		
Under bachelor’s degree	214	16.6	588	45.5	401	31	89	6.9	1292	100			0.03
Bachelor’s degree and higher	144	15	468	48.8	273	28.4	273	7.8	960	100			
Total	521	17	1437	46.9	892	29.1	211	6.9	3061	100			
Job												
Employee	250	15.4	771	47.4	476	29.2	131	8	1628	100		
Unemployed	156	23.2	295	43.9	177	26.3	44	6.5	672	100			<0.0001
Self-employee	110	15.1	353	48.6	229	31.5	35	4.8	727	100			
Total	516	17	1419	46.9	882	29.1	210	6.9	3027	100			
Age												
	N	Mean	SD	N	Mean	SD	N	Mean	SD	N	Mean	SD	P value
	526	34.8	14.4	1439	33.7	17.4	894	36.5	13.8	213	35	13.7	0.001

## 4. Discussion

The present study examined the sleep hygiene patterns and sleep hygiene behaviors of general population in Kermanshah, Iran. The greatest sleep hygiene problem was irregular sleep schedules (wake time and bedtime were different each day or wake time and bedtime were different during the weekend and holidays (74.8%)). Previous studies demonstrated an association between irregular sleep schedule and poor sleep quality (Carney, Edinger, Meyer, Lindman, & Istre, 2006; Kang & Chen, 2009; Monk et al., 2006). To date, only a few studies have directly reported the prevalence of irregular sleep schedules in the general population, but the prevalence of irregular sleep schedules in the current study was very high, and further research is needed to examine the impact of a fixed daily sleep and wake time and sleep time regularity during the weekend and holidays.

The next major sleep hygiene problem was day-time naps lasting for >2h (71%). Day-time napping has been proposed to disrupt the homeostatic sleep drive (Irish, Kline, Gunn, Buysse, & Hall, 2015), and some studies recommend avoiding naps for >30 min (Stepanski & Wyatt, 2003). Regular napping is a routine behavior for many Iranian people, as also indicated in the studies by Dhand et al. and Takahashi et al. (Dhand & Sohal, 2006; Takahashi, 2003). Whether persons who habitually indulge in day-time napping are more vulnerable to nocturnal sleep problems remains to be known. Individual and cultural differences may influence the consequences of day-time napping on nocturnal sleep. More importantly, in this population, prolonged habitual napping (>2 h) may be associated with higher morbidity and mortality. However, further investigation would be required to determine if avoidance of prolonged naps may improve sleep in the general population.

Regular exercise is an important and commonly recommended for sleep hygiene, but exercise should be avoided close to bed time (Stepanski & Wyatt, 2003). Based on the current findings, 60.7% of the general population did not have a regular exercise program during the week. Few studies have assessed the effect of exercise training on sleep in healthy populations. Awad et al. reported that exercise reduced the incidence of sleep-disordered breathing (Awad, Malhotra, Barnet, Quan, & Peppard, 2012).

Other major sleep hygiene problems were watching television in bed (55.3%), reading in bed (35.6%), talking on the phone (33.3%), and drinking caffeinated beverages (21.8%). In conclusion, only6.9% of all participants had good sleep hygiene, whereas 64.2% of participants had poor or very poor sleep hygiene. One reason may be the median age of participants (35 year) and some related factors (e.g. kids, an ill person in the family that needs care). Malakoti et al. showed that 82.6% of men and women, aged 60 years or older suffered from poor sleep quality (Malakouti, Foroughan, Nojomi, Ghalebandi, & Zandi, 2009) compared with 56% of Americans, 31% of western Europeans, and 29% of Japanese (Leger, Poursian, Neubouer, & Vchiyama, 2008). The higher prevalence of sleep disorders in Malakoti et al. study may be due to the number of elderly participants, but another reason based on our study may be poor sleep hygiene in Iran.

In the present study, sleep hygiene showed significant difference with respect to sex. Poor sleep hygiene was seen in men more than women. Evidence shows that the consumption of alcohol, cigarettes, and caffeine causes sleep hygiene disorder (Irish et al., 2015; Storfer-Isser, lebourgeois, Harsh, Tomposett, & Redline, 2013). The fact that in Iran consumption of such products is more prevalent among men than in women may be the reason for the significant difference with respect to sex.

There was a significant difference in sleep hygiene with respect to job. The current findings showed that unemployed people had poor sleep hygiene, which may be because of the work rules of on-time presence for employees.

The results also revealed that age factored a significant difference in sleep hygiene. As previously mentioned younger people had poor sleep hygiene, which may be because of the fact that they are not trained sufficiently or most of the younger people do not have a job and obligation for wake up in the morning. Nowadays the role of media and mobile phone and virtual social networks in younger peoples is important.

There was also a significant difference in sleep hygiene with respect to the level of education. The higher education levels are associated with higher sleep hygiene. For instance, Cho et al. found that the use of TV in bed room can be decreased by increasing education level, which can in turn have an impact on sleep hygiene (Cho, Kim, & Lee, 2013).

As conclusion we can say that poor sleep hygiene were more frequent in Iranian population and the major problem in sleep hygiene in our study was inappropriate sleep schedule including instability bed and wake time and daily napping more than 2 hours.

## 5. Strengths, Limitations, and Considerations

To the best of our knowledge, this is the first study to assess sleep hygiene using a large sample size in Iran and investigate a large number of factors that may be associated with adult sleep hygiene. The study was, however, restricted to only Kermanshah in Iran.
